# Mafeking

**Published:** 1900-05-26

**Authors:** 


### The Hospital

A Journal of

The Medical Sciences and Hospital Administration.

Vol. XXVIII.—No. 713.] Edited by Sir Henry Burdett, K.C.B., and Solomon C. Smith, M.D., M.R.C.P. [May 26, 1900.

### MAFEKING.

The depth and intensity of feeling which may lie concealed beneath the ordinarily tranquil and restrained demeanour of the English people has just been illustrated in a manner which may not impossibly serve to explain to some of our foreign critics a variety of circumstances by which they are apt to be perplexed, and to give them some notion of the latent power of which this depth and intensity are sources, and which any who assume positions hostile to us must, sooner or later, be prepared to reckon with. After forty-eight hours of almost delirious rejoicing, we again return quietly into our accustomed grooves, and display our habitual indifference to the acts and opinions of our self-constituted censors. The war, however it may be conducted or prolonged, or however rapidly the resistance of our enemies may find its natural termination in collapse and flight, seems no longer to offer any possibility of a disaster which no foresight on the part of our rulers could prevent, and of which the moral effects would be of far greater importance than the material. We shall now be content with the knowledge that it can have but one end, and that it has already practically abolished the distinction between the mother country and her colonies. The political blundering which permitted the severance from Great Britain of the United States has had for its chief consequence to defer for a century the pre-eminence of the Anglo-Saxon race throughout the world, but is at least partially redressed by that renewed and completed union among ourselves which might still have been long delayed had not the natural course of events been hastened to maturity by the folly and treachery of the Boers.

With general political considerations, however, although no Englishman can either be indifferent to them, or free from some share of responsibility as to the manner in which they are dealt with, we, in these columns, have no special concern; and, after the first burst of pride and exultation has had its way, we may turn our thoughts towards the heroes of Mafeking with a desire to know more than has at present been disclosed with regard to the material basis and the sanitary or insanitary consequences of their endurance. It is obvious that no reliance can be placed upon the accounts of the provisioning of the garrison which were made public during the siege, because these, although they may have been accurate, may equally have been designed to mislead the enemy. If the positions of the garrison and the besiegers had been reversed, there can be little doubt that the place would have been carried by assault months ago; and it is quite conceivable that accounts of shortness of provision may have been designedly put in circulation for the very purpose of acting upon the respectful consideration which the Boers have invariably shown for their own miserable skins, and of inducing them to wait for what they considered inevitable, and to refrain from attacks which it might not have been possible to repel. We may not impossibly find that the medical officers of the garrison have been witnesses of an experiment in dietetics of great and far-reaching interest, in which the two elements of insufficiency of quantity and unsuitability of quality may require to be carefully distinguished from each other. We fear there can be no question that there has been great prevalence of enteric fever and dysentery, as well as of other forms of disease; but the fever and the dysentery would presumably be mainly spread through the agency of polluted water, although the mortality which they would occasion would be greatly increased by the impossibility of obtaining a suitable dietary for the sick. Putting the sick aside, there is a widely-diffused belief among medical men that healthy people in comfortable circumstances habitually consume not only more food than they require, but more than is good for them; and the effects upon the healthy of a restricted diet continued over a sufficient period of time, and not complicated, as it would be in a prison, by a variety of depressing mental influences, will surely afford valuable data with regard to many questions connected with bodily nutrition. It is at least certain that, so recently as on the Saturday preceding the termination of the siege, the garrison were in sufficiently good fighting trim to kill and capture a considerable number of their assailants; and it will be important to know on what quantity and description of food this trim had been so successfully maintained. We shall wait anxiously for the details, not only as affording important facts with regard to the maintenance of bodily and mental vigour under conditions of exceptional hardship and trial, but also as an assistance in bestowing honour where honour is due. It will not improbably be found that the non-combatants of Mafeking have been little less worthy of imperial recognition than the brave and resourceful leader, or the gallant comrades and followers by whose aid his work has been accomplished.

